# Surgical Management of Primary Thoracic Epidural Melanoma

**DOI:** 10.7759/cureus.54536

**Published:** 2024-02-20

**Authors:** Taha Khalilullah, Giancarlo Mignucci-Jiménez, Henry Huffman, Hasita Karthikeyan, Zaheer Hanif, Ogechukwu Ariwodo, Ripul R Panchal

**Affiliations:** 1 Neurosurgery, American Neurospine Institute, Plano, USA; 2 Neurosurgery, Loyal and Edith Davis Neurosurgical Research Laboratory, Barrow Neurological Institute, Phoenix, USA; 3 Pathology, Ameripath North Texas, Plano, USA; 4 Neurosurgery, University of Texas Medical Branch, Galveston, USA; 5 Neurosurgery, Philadelphia College of Osteopathic Medicine South Georgia, Moultrie, USA

**Keywords:** spinal oncology, carbon-fiber peek screws, robotic-assisted navigation, case report, spinal epidural melanoma

## Abstract

In this study, we reported one of the first cases where a rare robotic-assisted platform with neuronavigation technology and carbon-fiber-polyetheretherketone (CF/PEEK) screws is employed to surgically treat multilevel thoracic primary spinal epidural melanoma. A 67-year-old male presented with left upper thoracic pain. His magnetic resonance imaging (MRI) of the thoracic spine revealed a dumbbell-shaped left epidural mass at the T2-3 level. Partial resection was performed due to tumor growth into the vertebral bodies and patient discretion for minimal surgery. The patient’s neurological conditions improved postoperatively, with reduced reported symptoms of pain and numbness. Postoperative imaging showed evidence of appropriate spinal stabilization. Patient underwent stereotactic body radiation therapy (SBRT), and no adverse events were reported. This case reflects one of the first examples of treating thoracic epidural melanoma with the use of robotic-assisted navigation. Further prospective studies are needed to determine the efficacy of robot-assisted navigation for patients with primary spinal malignant melanoma which may open the possibility of surgery to once presumed non-operative patients.

## Introduction

Melanoma is notoriously known as one of the most aggressive forms of skin cancer. However, melanoma is not limited to the skin, as melanocytes, the offending agent, exist in the uvea, cerebral parenchyma, leptomeninges, mucous membranes, and skin [1]. As such, melanocytic lesions can originate, or in other words, be a primary lesion, from either the dura, brain, or spinal cord. In fact, primary central nervous system (CNS) melanoma represents approximately 1% of all melanoma cases [2]. Primary spinal melanoma represents an even smaller percentage of melanoma cases. Most of them are intradural with or without extradural components [3]. Only 11 patients with primary spinal melanoma have been reported in the current literature, demonstrating the rarity of this particular neoplasm subtype and the lack of data regarding the standard treatment. However, a recent development in robotic-assisted spine surgery in which a rigid robotic arm is combined with neuronavigation technology has possibly opened the way for safer and more accurate procedures.

Robotic surgical platforms have gained popularity in oncological surgery because they can perform precise maneuvers even when anatomy is not entirely exposed [4-6]. Recent studies have demonstrated the ability of spinal navigation systems to reduce screw placement time, improve hardware implantation accuracy, and decrease the overall risk of reoperation and/or recurrence in comparison to free-hand techniques [4-9]. Even with peer-reviewed publications stating its utility and potential, robotic-assisted systems for spinal tumors continue to be an underexplored aspect of this technology. Here, we report one of the first cases where multilevel thoracic primary spinal epidural melanoma is managed surgically with a novel robotic-assisted platform that combines a robotic arm with neuronavigation technology and advanced carbon fiber-polyetheretherketone (CF/PEEK) screws.

## Case presentation

**Figure 1:**
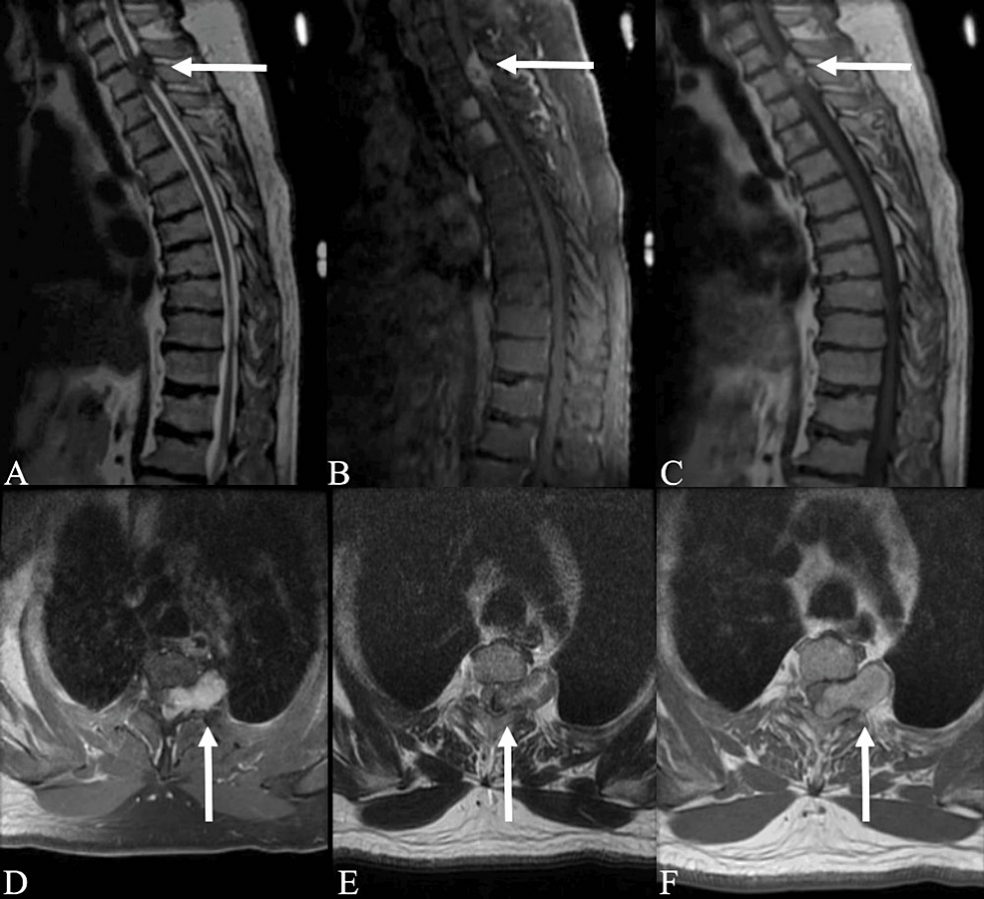
Preoperative MRI reveals ventral T2-3 left dumbbell-shaped spinal epidural melanoma involving thoracic cavity. The lesion appears hyperintense on sagittal and axial T1-weighted imaging (A: sagittal; D: axial) and hypointense on sagittal and axial T2-weighted imaging (B: sagittal; E: axial). Gadolinium-enhanced MRI reveals homogenous enhancement for the melanoma lesion (C: sagittal; F: axial).

A 67-year-old male who presented with a history of bitemporal headaches and severe and progressive left upper thoracic pain was recently found to have a mass at the T2-3 spine level. The patient has a past medical history of myasthenia gravis, hyperlipidemia, and no previous history of cancer. Neurological examination did not identify any neurological deficits or motor weakness, with cranial nerves II-XII grossly intact. Furthermore, deep tendon reflexes were 2+ in all extremities. Magnetic resonance imaging (MRI) of the thoracic spine revealed a dumbbell-shaped left epidural mass at the T2-3 level causing severe spinal cord compression and involvement of the thoracic cavity (Figure 1, panels D-F). In addition, contrast enhancement at the T3 and T4 vertebral bodies was noted. The mass was composed of hyperintense elements on T1-weighted images and hypointense elements on T2-weighted images (Figure 1, panels A-F).

Surgery

**Figure 2:**
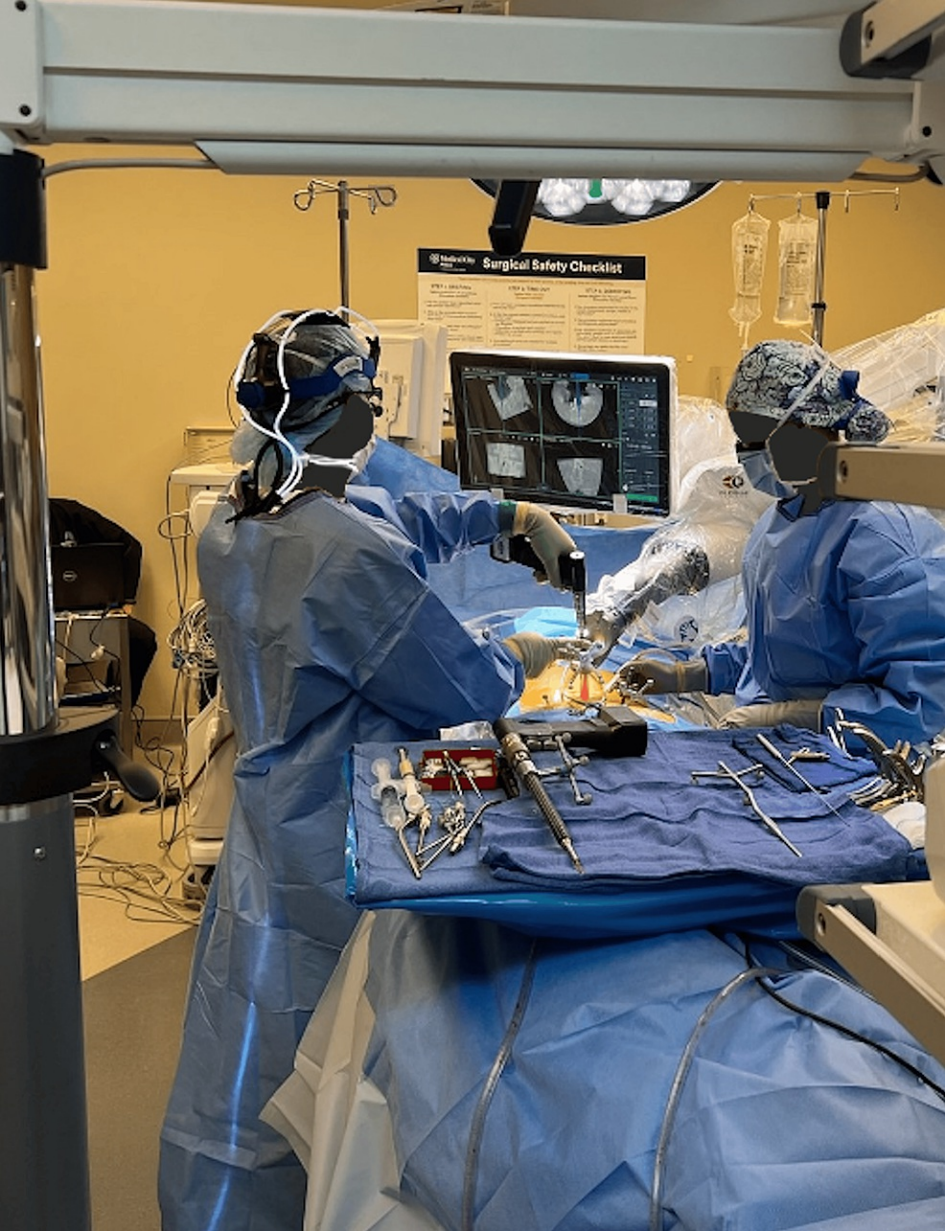
Surgeon employs Globus Excelsius robot-assisted navigation system (ExcelsiusGPS; Audubon, PA: Globus Medical, Inc.) to insert bilateral carbon-fiber/PEEK screws for proper stabilization. PEEK: polyetheretherketone

**Figure 3:**
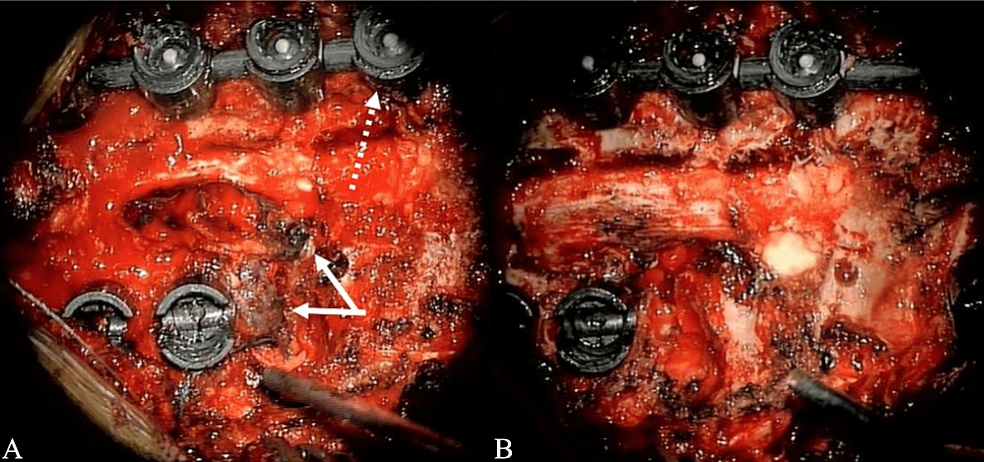
Intraoperative findings identified (A) black epidural melanoma that extends from T2 to T4 into the left lamina and pedicles and (B) the spinal cord is decompressed following partial tumor resection.

The patient underwent posterior decompression at the T2-4 levels. Additionally, non-total mass resection was performed with T1-5 arthrodesis with allografts and T2-3 left costotransversectomy for lateral mass resection. A midline incision was utilized with subperiosteal dissection performed to the lateral portion of the transverse processes bilaterally from T1-5. With the Globus Excelsius robot-assisted navigation system (ExcelsiusGPS; Audubon, PA: Globus Medical, Inc.), carbon-fiber/PEEK screws were placed bilaterally in T1-5 (Figure 2). Carbon-fiber/PEEK screws were employed due to the need to perform imaging in the future for monitoring metastatic spread and/or recurrence. Proceeding with decompression, the surgeon performed a T2-4 laminectomy and medial facetectomies. After the left T2-3 costotransversectomy, a dark epidural lesion was identified, extending from T2 to T4 and invading the left lamina and pedicles. Significant adhesions to the dura were noted, along with significant involvement of the nerve root at the left T2/3 level requiring its sacrifice. As demonstrated in the preoperative imaging, severe spinal stenosis was noted intraoperatively. Sufficient tumor resection from the dura was achieved, resulting in decompression and relaxation of the thecal sac (Figure 3, panels A and B). At the nine-month postoperative period, the bridging trabecular bone was <50% of AP distance and was indicated on standard AP x-rays.

Pathology

**Figure 4:**
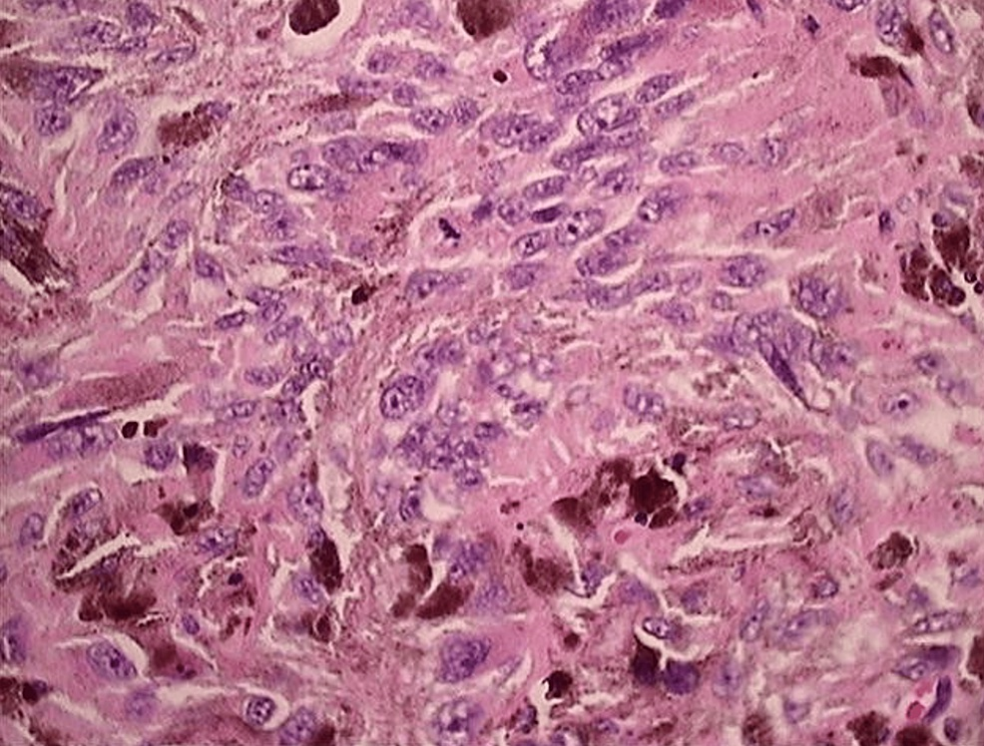
Histology of the primary spinal melanoma. (A and B) Immunohistochemical stains (1000x and SOX10, S100, and pan-melanoma stain and negative staining with pan-cytokeratin AE1/AE3) revealed irregular and enlarged pleomorphic nuclei with prominent nucleoli, vesicular chromatin, and occasional pseudo inclusions. Atypical mitotic figures present with involvement by a malignant neoplasm comprised of sheets of cells with epithelioid and spindle cell features.

Permanent specimens of the neoplasms were sent for pathological and immunohistochemical analysis. In addition, biopsies of the T3/4 vertebral body were undergone. Microscopic examinations revealed soft tissue and bone fragments, including a malignant neoplasm composed of sheets and nests of cells with epithelioid and spindle cell features. The cells exhibited abundant eosinophilic cytoplasm and contained pleomorphic nuclei, i.e., irregular and enlarged nuclei with prominent nucleoli, vesicular chromatin, and occasional pseudo inclusions (Figure 4, panels A and B). Additionally, scattered mitoses were presented with an atypical mitotic figure.

Immunohistochemical analysis revealed positive staining with SOX10, S100, and pan-melanoma stain and negative staining with pan-cytokeratin AE1/AE3. BRAF analysis was negative for the presence of the BRAF mutation (Figure 4, panels A and B). The morphologic findings were consistent with those of malignant melanoma.

Postoperative course

The patient’s neurological conditions improved postoperatively, with minimal reported symptoms of pain and numbness. Postoperative imaging showed evidence of appropriate spinal stabilization. After one month postsurgery, the patient underwent five fractions of 500 cGy stereotactic body radiation therapy (SBRT). There were no adverse events reported with radiation therapy administration, and the patient responded well to the treatment.

Careful postoperative examinations for a primary source elsewhere in the body did not discover dermatologic, gastrointestinal, or ophthalmologic lesions. Positive emission tomography (PET) scans discovered moderate uptake at T3 and T4, mild diffuse intake in surrounding soft tissue indicative of inflammation, and no other suspicious uptake elsewhere. As a result, the patient was diagnosed with primary thoracic epidural melanoma.

## Discussion

Comparison of melanomas

The location and presentation of cranial and spinal melanomas are the main differences between these two types of melanomas. Whereas spinal melanoma causes symptoms like back discomfort, paralysis, or numbness because of spinal cord compression, cranial melanoma usually appears as pigmented lesions on the scalp, face, or neck. Even though they are both aggressive tumors, spinal melanoma frequently has a worse prognosis because of treatment difficulties and possible neurological side effects. Surgery, radiation therapy, and chemotherapy are the usual course of treatment; in more advanced cases, targeted therapy or immunotherapy may be used. Effective management of these illnesses depends on early discovery and timely medical intervention.

Workup of primary spinal melanoma

The symptoms of primary spinal melanoma (PSM) are non-specific and commonly entail back pain and myelopathy indicated by unilateral spinal cord compression of nerve root [10-15]. Other common symptoms include weakness, paresthesia, and radicular pain [16-23]. MRI remains the gold standard for preoperative diagnosis of spinal cord melanoma [2]. On imaging, melanoma reveals hyperintense and hypointense components on T1 and T2 weighted sequences, respectively, due to the paramagnetic effect of melanin and hemorrhaged foci, hemorrhage causes intermediate signals in melanoma, while increased melanin results in higher intensity on T1-weighted images and lower intensity on T2-weighted images [16,24]. After the addition of contrast, the melanoma manifests as a homogenous enhancement. However, the appearance of melanoma on MRI varies significantly depending on the degree of melanin and hemorrhage [2,3,24]. Therefore, it is difficult to distinguish melanoma from other spinal cord tumors, and an exact diagnosis cannot be solely on MR imaging [25]. In the present case, the MR imaging corresponded with a diagnosis of malignant melanoma, but additional confirmation with biopsy and histopathological analysis was required.

The Hayward criteria is a classification system introduced in 1976 to help diagnose primary melanoma of the CNS. The present case meets the criteria with the following: (1) absence of malignant melanoma outside of CNS and (2) histopathologic testing for melanoma [26,27]. While patients with primary malignant melanoma have experienced extended survival time and/or cure, patients with metastatic melanoma of the CNS have had a significantly diminished prognosis [2,25,28-34]. On average, individuals with PSM live six years longer than patients with metastatic melanoma in the CNS [31]. Consequently, it is critical to determine whether the tumor is primary or secondary (i.e., metastatic). Common sites to consider for the origin of a metastatic melanoma lesion are skin, eyes, and GI tract [16,35]. Exclusion of lesions in these locations is determined with careful dermatological and retinal examinations, gastrointestinal endoscopies, and CT scans [36].

A histopathological analysis must be conducted to diagnose PSM. Histological features of primary melanoma include large cells with irregular nuclei, positive immunostaining of S100, HMB-45, and vimentin, and a negative result of the epithelial membrane antigen [20,37]. Additional indicators of PSM are cytoplasmic pigment, high mitotic rate with necrosis, infiltrative pattern, and nuclear pleomorphism [16]. Here, clinical, radiological, and histological evidence were present, as a result, leading the physician to diagnose the lesion as primary spinal epidural melanoma of the thoracic spine.

Treatment of PSM

Treatment options for primary CNS tumors include radiation with and without chemotherapy, radiosurgery, hormonal therapy, and surgical decompression with radiation [38]. Following standard oncologic rules, complete surgical resection is recommended due to the aggressive nature of melanoma and its potential to cure the patient [29,39,40]. Importantly, complete resection leads to a longer survival time and improved outcomes compared to partial (i.e., subtotal) resection [2,19,25,29]. Yet, some reports in the literature claim no correlation between the degree of resection and overall survival-commenting that symptoms can be ameliorated with partial resection [20,31]. In this case, partial resection was performed due to tumor growth into the vertebral bodies and laminas, and patient discretion for minimal surgery. As such, radiotherapy was implemented postoperatively. However, no standard radiotherapy or chemotherapy regimen exists, and it is unknown whether either is effective for PSM. Furthermore, it is questionable whether PSM is radiosensitive to radiation therapy [2,31,41]. Certain studies claim that adjuvant therapy has controlled tumor growth and reduced metastasis, but did not impact overall survival time [20,40]. On the other hand, in a recent study by Kim et al., they found favorable outcomes without adjuvant therapy [25]. Despite clear evidence, most physicians recommend surgical resection with postoperative adjuvant therapy for all patients, demonstrating the need for further investigation and clinical reports like the present case [2,29,31,33,35,42].

Surgical management: spinal instrumentation and technology

Instrumentation and fusion of the spine are sometimes required for surgical resection of a spinal tumor to maintain stabilization [23,43,44]. Traditionally, spinal stabilization is achieved with titanium implants, such as pedicle screws and rods [45]. However, instrumentation made of titanium causes artifacts on MRI and CT scans. These artifacts are counterproductive for stabilization after surgery for spinal tumors because they prevent tumor progression monitoring and inhibit conventional radiation planning, accuracy, and delivery [46]. The metal hardware alters the radiotherapy by causing a dose attenuation and absorption that shields the implanted area and irradiates surrounding tissues through backscattering [3,45,47-49]. To combat this, implants constructed of polyetheretherketone (PEEK) with carbon fiber were created. Notably, carbon fiber-PEEK screws, even if lacking flexibility, have proven to have equal stability/effectiveness and improved biological compatibility and radiation delivery compared to titanium implants [45,49]. Importantly, these constructs are also suitable for posterior upper thoracic fixation [23].

Robot-assisted surgery for spinal tumors

The advent of robotic technology combined with neuronavigation systems has introduced a novel method to treat spinal tumors. Recent research on instrumentation placement via robotic-assisted surgery has demonstrated improved pedicle accuracy, decreased radiation dosages, faster recovery, and diminished postoperative complications due to limited exposure [50-52]. Studies have revealed fewer proximal facet joint violations and decreased pedicle breaches compared to free-hand screw placement-ultimately minimizing neurovascular injuries and construct failures [51-55]. Additionally, robot-assisted navigation platforms have successfully planned spinal tumor resections while reducing the degree of surgery [55-57]. However, robotic-assisted navigation in surgery is still in its infancy, and further prospective studies are required to confirm the effectiveness of this technology.

Robotic navigation is theorized to become an essential tool for spine tumor surgery due to the inherent difficulty of obtaining adequate surgical margins and the manipulation of normal anatomy. By visualizing the localization of the neoplasm with intraoperative navigation, surgeons can better identify the unexposed anatomy hidden by primary or metastatic lesions [55,58]. For example, in the present case, a dural injury was possible due to epidural detachment of the melanoma. However, with navigation and real-time feedback, the surgeon could create a perioperative plan for the desired resection with sufficient vertebral resection. Additionally, following decompression and resection, subsequent stabilization was confidently achieved due to the robotic-assisted navigation technology, which provided optimal trajectory for hardware placement at all spine levels [53,59]. This report agrees with a recent study on computer-assisted navigation that demonstrated adequate resection of compressive thoracic tumors and symptomatic improvement [60-62]. Although similar findings with robot-assisted navigation have not yet been described, the study demonstrates the promising prospect of robotic-guided operations.

With paravertebral and paraspinal en bloc resections, current reports have exemplified the effectiveness of robot navigation by enabling a multidisciplinary approach, resection of adjacent neoplasms, and preservation of normal tissue [63-65]. Nonetheless, research on robot-guided navigation and resectioning epidural spine melanoma is still limited. Further studies are needed to recognize the value of robotic-assisted navigation in primary thoracic epidural melanoma cases.

Discussion summary

Melanoma is a rare malignant neoplasm of melanocytes (i.e., melanin-producing cells) arising from the neural crest during embryogenesis that migrates to the skin, mucous membranes, and CNS. Primary spinal melanoma (PSM) is uncommon, constituting less than 1% of all melanoma cases, with 90% being metastatic in nature [2,7-9]. The exact cellular origin of PSM is unknown, while some believe it may derive from neuroectodermal cells or melanocytes in the leptomeninges at the anterior and lateral portions of the spinal cord and brainstem [10,11]. The melanoma in this report may originate from the anterior meninges. To the best of our knowledge, this report is the first to demonstrate the use of a robotic-assisted platform for primary melanoma of the spine. Importantly, this case will add another clinical example of PSM, where only 11 cases of PSM are found in the current literature, of which four cases are reported to be in the epidural space [3,8,12-15].

## Conclusions

We report, for the first time, a case where surgical management of a rare multilevel thoracic primary epidural melanoma was achieved with robotic-assisted navigation and advanced carbon fiber-PEEK implants. Partial resection could only be achieved and radiotherapy was implemented one month postoperation. With primary malignant melanoma, careful examination of patient history, imaging findings, and histopathological analysis are required to attain an accurate diagnosis. Unfortunately, due to its rare incidence and unpredictable clinical course, a standardized chemotherapy and radiotherapy regimen has not been established for primary spinal cord melanoma. However, with advances in robotic-assisted surgery, surgeons can identify and develop efficient methods to enable precise surgical resection of spinal tumors and approach deformed spinal anatomy with minimal complications. Further prospective studies that determine the efficacy of robot-assisted navigation are necessary to improve outcomes for patients with primary spinal malignant melanoma and open the possibility of surgery to once presumed non-operative patients.
